# Activités physiques libres ou encadrées et condition physique liée à la santé chez des adultes burundais: étude transversale

**DOI:** 10.11604/pamj.2016.25.38.7688

**Published:** 2016-09-28

**Authors:** Jean Berchmans Bizimana, Mansourou Mohamed Lawani, Barnabé Akplogan, Charles Gaturagi

**Affiliations:** 1 Laboratoire de Biomécanique et Performance (LABIOP), Institut National de la Jeunesse, de l’Education Physique et du Sport (INJEPS), Université d’Abomey-Calavi, 01 BP 169 Porto-Novo, Bénin; 2 Institut d’Education Physique et des Sports, Université du Burundi, BP 1500 Bujumbura, Burundi

**Keywords:** Activité physique, condition physique liée à la santé, santé, Burundi, Physical activity, health-related physical fitness, health, Burundi

## Abstract

**Introduction:**

l’activité physique régulière a un impact positif sur la santé. Cette étude a pour objet de comparer la condition physique liée à la santé des adultes qui s’exercent librement avec celle des adultes bénéficiant d’un encadrement professionnel. Elle tente aussi d’établir une relation entre le niveau d’activité physique et les paramètres de la condition liée à la santé.

**Méthodes:**

nous avons évalué le niveau d’activité physique et les paramètres de la condition physique liée à la santé. Par le test t pour échantillons indépendants, nous avons comparé les moyennes et avons par le calcul du coefficient de corrélation r de Pearson analysé la relation entre le niveau d’activité physique et les paramètres de la condition physique.

**Résultats:**

des écarts significatifs (p < 0,05) de niveau d’activité physique, de souplesse, de ${\dot{\text{V}}\text{O}}_{\text{2}}\max$ et de la FC de repos ont été enregistrés en faveur du groupe encadré. Le niveau d’activité physique est positivement corrélé (p < 0,05) au ${\dot{\text{V}}\text{O}}_{\text{2}}\max$ et à la force de préhension mais négativement corrélé à la FC de repos et au cholestérol LDL. La prévalence des facteurs de risque cardiovasculaire n’est pas élevée excepté pour le cholestérol HDL.

**Conclusion:**

les résultats de cette étude montrent que l’activité physique libre est aussi efficace que l’activité physique encadrée dans le maintien des profils lipidique et physiologique favorables à la santé chez l’adulte burundais. Cependant, l’activité physique encadrée apporte des bénéfices supplémentaires pour le ${\dot{\text{V}}\text{O}}_{\text{2}}\max$, la fréquence cardiaque de repos, la souplesse antérieure et la détente verticale

## Abstract (en)

**Introduction:**

Regular physical activity has a positive impact on health. This study aims to compare health-related physical fitness in adults engaged in organized physical exercise with these in adults engaged in nonorganized physical exercise and to establish a relationship between this physical fitness and physical activity level.

**Methods:**

We collected data on physical activity level and health-related physical fitness parameters. Independent samples T-test was used to compare the means of the two groups and Pearson correlation coefficient calculator was used to analyze the relationship between physical fitness parameters and physical activity level.

**Results:**

Significant differences (p < 0.05) in physical activity level, flexibility, aerobic endurance (${\dot{\text{V}}\text{O}}_{\text{2}}\max$) and HR rest were recorded for the organized exercise group. The level of physical activity was positively correlated (P < 0.05) to ${\dot{\text{V}}\text{O}}_{\text{2}}\max$ and grip strength but it was negatively correlated with HR rest and LDL cholesterol. The prevalence of cardiovascular risk factors was minimal except for HDL cholesterol.

**Conclusion:**

The results of this study show that nonorganized physical exercise is as effective as organized physical exercise in maintaining proper health-related physiological and lipid profiles in Burundian adult. However, organized physical activity provides additional health benefits on ${\dot{\text{V}}\text{O}}_{\text{2}}\max$, HR rest, anteroposterior flexibility and vertical jump.

## Introduction

L’effet bénéfique de l’activité physique régulière sur la santé est bien connu depuis l’antiquité. Néanmoins, ces dernières décennies, les conceptions sur l’activité physique et la santé ont beaucoup évolué et la promotion de l’activité physique comme mesure préventive en santé publique a gagné en importance [1, 2]. Selon l’OMS, la santé est un « état de bien-être physique, mental et social, qui ne consiste pas seulement en une absence de maladie ou d'infirmité [3]. Cette définition est apparentée à celle de la condition physique qui est la « capacité de mener à bien les tâches de la vie quotidienne, avec vigueur et vigilance, sans fatigue indue, et avec une ample réserve d’énergie permettant de pouvoir jouir de ses loisirs et de pouvoir faire face aux situations critiques et imprévues » [4]. S’appuyant sur les relations connues entre la condition physique et la santé, Pate [5] a distingué la condition physique liée à la santé de la condition physique liée à la performance. La condition physique liée à la santé se réfère aux caractéristiques physiques et physiologiques qui définissent les niveaux de risque de développement prématuré des maladies ou des conditions morbides présentant un rapport avec un mode de vie sédentaire [6]. Un certain nombre de composantes mesurables contribuent à son intégralité à savoir la composante morphologique, la composante musculaire, la composante motrice, la composante cardiorespiratoire et la composante métabolique [6].

*Il est admis que l'exercice physique a une influence directe sur la majorité de ces composantes dont plusieurs sont considérées comme des précurseurs de la maladie et identifiées comme des facteurs de risque en recherche clinique [7]. En effet, lorsque l’activité physique est pratiquée à une intensité, une durée et une fréquence appropriées, le corps répond par une série de changements positifs intégrés dans le fonctionnement de presque tous ses systèmes. La santé résulte donc des réponses adaptatives à l'effort régulier exercé sur divers tissus et fonctions biologiques par les demandes métaboliques, physiques ou mécaniques accrues [8]. Actuellement, il a été établi l’existence d’une relation dose-réponse inverse le plus souvent linéaire, entre le volume d’activité physique et le risque de mortalité toutes causes, le risque de maladies cardiovasculaires, l’hypertension et le risque de diabète de type 2 [9–11]*.

Au Burundi, des études de dépistage ont montré une évolution inquiétante des maladies liées au mode de vie sédentaire chez la population urbaine adulte [12, 13]. Cependant, nous observons ces dernières années de nombreuses personnes qui, en dehors de leur temps de travail s’adonnent à diverses activités physiques et sportives sous la forme encadrée ou non encadrée. Ce qui pourrait être un atout dans la lutte contre ces maladies surtout qu’une étude très récente a montré que la recherche d’un bon état de santé était citée par cette population comme principale motivation de pratique [14]. Malheureusement, nous ne voyons pas s’impliquer l’autorité ou tout autre organisme dans l’encadrement effectif de ces activités ou l’évaluation de leur impact. Ce qui peut être préjudiciable à l’aboutissement aux résultats escomptés notamment l’amélioration ou le maintien d’une bonne condition physique.

*En effet, si dans la pratique d’activité physique non encadrée, les individus ont l’avantage du coût réduit et de l’autonomie par rapport à l’horaire et au lieu de pratique, ils sont parfois irréguliers et peuvent se fixer des objectifs inadaptés à leur condition physique ou mal gérer leur entraînement. Ce qui peut constituer un obstacle à une pratique efficiente bien que la littérature ait rapporté des résultats intéressants pour cette pratique dans l’amélioration des paramètres de la condition physique liée à la santé [* *15* *]. Cependant, la pratique encadrée semble induire des effets bénéfiques supplémentaires [* *16* *,* *17* *]*.

L’objet principal de cette étude est d’analyser l’impact de la pratique de l’activité physique d’entretien, surtout la pratique populaire spontanée (non encadrée) sur la condition physique liée à la santé chez les adultes burundais. Nous comparons d’une part, le niveau d’activité physique et d’autre part la condition physique liée à la santé des adultes qui s’exercent librement avec ceux des adultes bénéficiant d’un encadrement professionnel. Ensuite, nous recherchons la relation entre le niveau d’activité physique et les paramètres de la condition liée à la santé.

## Méthodes

### Type d’étude et cadre de réalisation

Il s’agit d’une étude transversale et analytique, effectuée en septembre 2014 au Laboratoire de Biomécanique et Performance (LABIOP) de l’Institut National de la Jeunesse, de l’Education Physique et du Sport (Benin) et à l´Institut d’Education Physique et des Sports (IEPS) au Burundi (Afrique Centrale).

### Population et échantillonnage d’étude

La population d’étude était composée d’adultes qui pratiquaient les activités physiques d’entretien. Deux catégories ont été visées à savoir une catégorie qui bénéficiait d’un encadrement professionnel et une autre de pratiquants spontanés. Au total, 103 sujets avaient manifesté la volonté de participer à l’étude mais seuls 90 sujets y ont pris part: 47 sujets qui s’exerçaient en pratique encadrée et 43 sujets qui s’exerçaient dans un cadre libéral.

Critères d’inclusion: pratiquer une activité physique d’entretien au moins une fois le mois; avoir un âge compris entre 18 et 65 ans; être volontaire et avoir signé un consentement éclairé.

Critères de non inclusion: être âgé de moins de 18 ans et de plus de 65 ans; être sous traitement médicamenteux.

Critères d’exclusion: rater au moins trois tests.

**Protocole expérimental**: il s’est déroulé en quatre étapes:

**visite des lieux de pratique:** cette visite nous a permis de rencontrer les sportifs et de les informer des objectifs de l’étude afin d’obtenir leur consentement éclairé dans le respect de la Déclaration d’Helsinki (1964).

**Choix de l’outil d’évaluation du niveau d’activité physique habituelle:** le niveau d’activité physique a été évalué à l’aide du questionnaire de Baecke [18]. Ce questionnaire auto-administrable se compose de seize questions et permet de déterminer trois indices représentatifs de l’activité physique habituelle: un indice d’activité de travail (IAT, huit questions), un indice d’activité sportive (IAS, quatre questions) et un indice d’activité de loisir (IAL, quatre questions). Il a été choisi en raison de son évaluation positive dans les études antérieures [19].

**Enregistrement de l’âge et des paramètres anthropométriques:** le poids et la taille ont été mesurés respectivement à l’aide d’un pèse personne électronique QE 2003A précis à 1/10ème avec une portée maximale de 150 kg et d’une toise de marque Fazzini S225. Le poids a été enregistré à 0,1 kg près tandis que la taille a été mesurée au 0,1cm. L’indice de masse corporel (IMC) a été calculé selon la formule suivante: masse (kg) divisé par la taille (en mètre) au carré.

### Evaluation des paramètres musculaires et cardiorespiratoires

La flexibilité postéro-antérieure et la détente verticale ont été respectivement évaluées à l’aide du flexomètre avant TK500 et du jumpmètre TK200 tous de fabrication japonaise. Trois essais étaient accordés à chaque sujet et le meilleur essai exprimé par le nombre de centimètres affichés sur l’écran de l’appareil était enregistré. La force de préhension a été évaluée à l’aide d’un dynamomètre TKK 5401 de fabrication japonaise. Deux essais étaient exécutés alternativement sur chaque main et la moyenne des meilleurs essais de chaque main était enregistrée à 0,5 kg près. Le ${\dot{\text{V}}\text{O}}_{\text{2}}\max$ a été prédit à l’aide de la course navette de Léger. C’est un test valide pour prédire le ${\dot{\text{V}}\text{O}}_{\text{2}}\max$. En fonction de l’âge des sujets, il présente un niveau de corrélation compris entre 0,70 (n = 188 enfants) et 0,90 (n = 77 adultes) [20].

### Evaluation des paramètres cardiovasculaires et métaboliques

La pression artérielle et la fréquence cardiaque de repos ont été mesurées en utilisant respectivement un système numérique automatique (Omran Digital Hem-907, Tokyo) et des cardiofréquencemètres de marque Polar après 5 à 10 minutes de repos. Trois lectures ont été prises pour chaque participant et une moyenne de ces lectures a été employée dans l’analyse finale. Les échantillons de sang veineux ont été collectés par deux infirmiers le matin à l’arrivée du participant après au moins 8 heures de jeûne. Tous les échantillons ont été analysés au Laboratoire d’Analyse Médicale de Bujumbura (LAMEB). Le cholestérol total (CT), les triglycérides (TG), les cholestérols HDL et LDL ainsi que le glucose à jeun ont été analysés par la méthode de calorimétrie enzymatique. Le Cholestérol total, le cholestérol LDL et le TG étaient considérés comme élevés pour des valeurs respectivement supérieures ou égales à 6,2 mmol/l, à 3,8 mmol/l et à 1,7mmol/l tandis que le cholestérol HDL était considéré bas pour une valeur inférieure à 1,0 mmol/l [21]. Le diabète était défini avec un niveau de glucose sanguin à jeun supérieur ou égal à 7,0 mmol/l [22].

**Analyse des données:** les données ont été analysées avec le logiciel SPSS (version 20.0). Les statistiques descriptives ont été calculées pour chaque variable étudiée. Par le test t pour échantillons indépendants, nous avons comparé les moyennes des variables des deux échantillons. Le calcul du coefficient de corrélation r de Pearson, nous a permis de rechercher la force d’association entre les paramètres de la condition physique et le niveau d’activité physique. Le niveau de significativité des tests a été fixé à p < 0,05.

## Résultats

L’âge moyen des sujets non encadrés était de 34,72 ± 7, 20 tandis qu’il était de 31,09 ± 5,94 chez les sujets encadrés. L’analyse des résultats n’a révélé aucune différence significative (p>0,05) entre les deux groupes pour les paramètres anthropométriques (Tableau 1). La comparaison des trois indices d’activité a montré un indice d’activité sportive (IAS) significativement élevé (p < 0,05) chez le groupe encadré. Aucune différence significative (p>0,05) n’a été enregistrée entre les deux groupe pour l’indice d’activité de loisir et l’indice d’activité de travail (Tableau 2). L’analyse des moyennes obtenues a révélé une souplesse antérieure significativement élevée (p < 0,05) dans le groupe encadré pour les paramètres musculaires. En ce qui concerne les paramètres physiologiques, nous avons remarqué une fréquence cardiaque significativement basse (p < 0,05) et un ${\dot{\text{V}}\text{O}}_{\text{2}}\max$ significativement élevée (p < 0,05) dans le groupe encadré (Tableau 3). Par contre, aucune différence significative (p > 0,05) n’a été observée pour les paramètres métaboliques excepté le LDL (p< 0,05). Les corrélations entre le niveau d’activité physique (mesurée par l’indice d’activité physique habituelle) et les composantes de la condition physique sont présentées dans le Tableau 4. Dans le groupe professionnellement encadré, l’activité physique présentait une relation positive significative avec la force de préhension (r= 0,42; p < 0,05), la détente verticale (r = 0,57; p < 0,05), le ${\dot{\text{V}}\text{O}}_{\text{2}}\max$ (r = 0,67; p < 0,05) et une corrélation négative avec la fréquence cardiaque de repos (r = - 0,51; p < 0,05). Aucune autre corrélation significative n’a été enregistrée entre le niveau d’activité physique et les composantes de la condition physique. La prévalence des facteurs de risque cardiovasculaire est présentée dans le Tableau 5. Aucune différence significative n’a été observée (p>0,05) entre ceux qui bénéficiaient d’un encadrement professionnel et ceux qui n’en bénéficiaient pas. La prévalence des facteurs de risque cardiovasculaire était relativement basse aussi bien dans le groupe encadré que dans celui non encadré excepté le HDL: 2,1% contre 9,3% pour l’hypertension, 19,15% contre 27,91% pour le cholestérol HDL bas, 0% contre 4,60% pour l’obésité générale, 4,3% contre 4,7% pour l’hypercholestérolémie, 6,38% contre 6,98% pour le cholestérol LDL élevé, 6,38 % contre 11,6% pour les triglycérides, et 4,26% contre 11,63% pour la glycémie à jeun.

**Tableau 1:** Caractéristiques anthropométriques des sujets

	sujets non encadrés	sujets encadrés	P
Age (années)	33,72 ± 7,20	31,09 ± 5,59	0,53
Taille (m)	1,71 ± 0,93	1,72 ± 0,89	0,42
Poids (kg)	69, 39 ±12,12	64,39 ± 7,78	0,09
IMC	23,56	21,95	0,06

IMC: indice de masse corporelle

**Tableau 2:** Niveau d’activité physique habituelle

	sujets non encadrés	sujets encadrés	p value
Indice d’activité de loisir	2,70 ± 0,31	2,74 ± 0,11	0,63
Indice d’activé de travail	2,43 ± 0,21	2,58 ± 0,54	0,06
Indice d’activité sportive	2,89 ± 0,36	3,18 ± 0,32	0,02^+^
Indice d’activité habituelle	8,02 ± 0,43	8,48± 0,49	0,01^+^

**Tableau 3:** Comparaison des moyennes ± écart-type

	sujets non encadré	sujets encadrés	valeur de P
**Capacités physiques**			
Force de préhension (en kg)	39,38 ± 9,03	38,05 ± 7,72	0,43
Souplesse (cm)	22,17 ± 9,11	26,53 ± 9,31^+^	0,03
Détente verticale (cm)	43,38 ± 10,32	47,65 ± 9,51^+^	0,04
${\dot{\text{V}}\text{O}}_{\text{2}}\max$ (ml/kg/min)	34,47 ± 4,72	39,45 ± 6,72^+^	0,00
Force de préhension (en kg)	39,38 ± 9,03	38,05 ± 7,72	0,43
**Paramètres physiologiques**			
PA systolique (mm Hg)	124,0 ± 11,35	120,32 ± 8,04	0,07
PA diastolique (mm Hg)	75,44 ± 7,99	73,78 ± 6,93	0,36
FC de base (bpms/min)	69,53 ± 7,06	65,04 ± 6,03^+^	0,03
**Paramètres métaboliques**			
Cholestérol total (mmol/l)	4,23 ± 1,01	4, 27 ± 0,81	0,85
Triglycérides (mmol/l)	0,98 ± 0,58	0,95 ± 0,52	0,83
Cholestérol HDL (mmol/l)	0,64 ± 0,39	0,68 ± 0,42	0,63
Cholestérol LDL (mmol /l)	1, 22 ±0,79	1,09 ± 0,66	0,42
Glycémie à jeun (mmol /l)	5,02 ± 0,89	4,93 ± 0,72	0,60

PA: pression artérielle; FC: Fréquence cardiaque; bpms: battements; HDL: High density liproprotein; LDL: Low density liproprotein

**Tableau 4:** Prévalence de risque cardiovasculaire dans le groupe encadré et le groupe non encadré

Facteurs de risque cardiovasculaire	sujets encadrés (47)	sujets non encadrés (43)	Total (90)
**Tension artérielle (PA ≥ 140/90)**			
Elevée	1(2,1 %)	4 (9,3 %)	5 (5, 6 %)
Normale	46 (97,9 %)	39 (90,7 %)	85 (94,4%
**Obésité générale (IMC≥30kg/m^2^)**			
Obèses	0 (00 %)	2 (4,6 %)	2 (2,22 %)
Non obèses	47 (100 %)	41 (95,4 %)	88(97,8 %)
**Cholestérolémie (≥ 6,2 mmol/L)**			
Elevée	2 (4,3%)	2 (4,7%)	4 (4,5 %)
Normale	45 (95,7%)	41 (95,3%)	86(95,5 %)
**Cholestérol LDL (≥ 3,8mmol/L)**			
Elevé	3 (00 %)	3 (00 %)	6 (6,67 %)
Normal	44 (93,62 %)	40 (93,02 %)	84 (93,33 %)
**Cholestérol HDL (<1,0mmol/L)**			
Bas	9 (19,15 %)	12 (27, 91 %)	21 (23,3 %)
Normal	38 (80, 85%)	31 (72,1%)	69 (76, 7%)
**Triglycérides (≥1,7mmol/L)**			
Elevé	3 (6,38 %)	5 (11,6 %)	8 (8,9 %)
Normal	44 (93,62 %)	38 (88,4%)	82 (91,1%)
**Glycémie à jeun (≥ 7,1mmol/L)**			
Elevé	2 (4,26%)	5 (11,63%)	7 (7,8%)
Normale	45 (95,74%)	38 (88,37 %)	83 (92,2 %)

**Tableau 5:** Corrélation entre le niveau d’activité physique et les composantes de la condition physique liée à la santé

	groupe non encadré		groupe engendré	
	r	p	r	p
**Paramètres physiologiques**				
PA systolique (mm Hg)	- 0,12	0,45	- 0,15	0,31
PA diastolique (mm Hg)	- 0, 23	0,14	- 0,03	0,85
Fréquence cardiaque de base	- 0, 05	0,76	- 0,51^+^	0,01^+^
**Capacités physiques**				
Force de préhension	0,05	0,74	0,42^+^	0,001^+^
Souplesse antéro-postérieure	0,17	0,57	0,08	0,58
Détente verticale	0,07	0,67	0,57^+^	0,001^+^
${\dot{\text{V}}\text{O}}_{\text{2}}\max$	0,06	0,72	0,67^+^	0,001^+^
**Paramètres métaboliques**				
Cholestérol total	- 0,12	0,43	- 0,17	0,27
Triglycérides	- 0,05	0,74	- 0,08	0,59
Cholestérol HDL	- 0,07	0, 64	- 0, 20	0,19
Cholestérol LDL	- 0,05	0,76	- 0, 29	0,05
Glycémie à jeun	0, 24	0,12	0,12	0,41

r: Coefficient de corrélation de Pearson; p) Seuil de significativité

## Discussion

La comparaison des indices d’activité de travail et des indices d’activité de loisir n’a pas révélé une différence significative (p>0,05) entre le groupe encadré et le groupe non encadré. Ce qui indique que les sujets de ces groupes auraient le même niveau d’engagement dans les activités physiques de loisir et que leurs activités professionnelles ne sont pas aussi différentes quant à leur exigence physique. Par contre, les résultats de cette étude ont montré un niveau d’activité physique significativement élevé (p < 0,05) chez les sujets qui bénéficiaient d’un encadrement professionnel, probablement du à une plus grande participation aux activités sportives. Ce qui expliquerait leur endurance aérobie (mesurée par le ${\dot{\text{V}}\text{O}}_{\text{2}}\max$) significativement élevée (p < 0,05) et leur fréquence cardiaque de repos (FCR) significativement basse (p < 0,05). En effet, l’entraînement physique augmente considérablement la taille du cœur et de ce fait, les ventricules qui, à leur tour améliorent l’activité contractile du cœur. Or, il a été montré que le débit cardiaque maximal est le facteur principal limitant le ${\dot{\text{V}}\text{O}}_{\text{2}}\max$ dans l’exercice de type aérobie [23]. Bien que toute forme d’activité physique d’intensité modérée améliore le débit cardiaque, l’entraînement d’intensité élevée et surtout de type athlétique augmente la taille et le volume systolique. Ce qui conduit à une amélioration du débit cardiaque de repos et par conséquent celle du ${\dot{\text{V}}\text{O}}_{\text{2}}\max$.

Les performances enregistrées en détente verticale et en flexibilité chez les sujets qui bénéficiaient d’un encadrement professionnel étaient bonnes une fois comparées à celles rapportées dans les études antérieures [24, 25]. La force de préhension était aussi comparable à celle mesurée sur les populations asiatiques [26] mais plus faible que celle rapportée chez les populations afro-caraïbes et européennes de même âge [27, 28]. Les raisons de ces différences sont multiples. La taille et le poids des sujets, la position du corps, les angles de flexion de l’épaule et de l’avant bras, les encouragements donnés par l’évaluateur, l’intervalle entre la prise des mesures et le temps de la journée sont autant de facteurs qui peuvent expliquer ces différences [29, 30]. Ces meilleures performances enregistrées dans le groupe encadré (p < 0,05) seraient le résultat d’une bonne prise en charge incluant le renforcement musculaire aussi bien que l’assouplissement musculaire et articulaire. En effet, alors que les sujets de ce groupe s’exerçaient sous la responsabilité d’un encadreur qui maitrise plus ou moins les règles d’une séance d’activité physique d’entretien, les sujets non encadrés n’étaient pas soumis à ces règles. En outre, la majorité des sujets encadrés ont déclaré jouer parfois au basketball ou au volleyball en dehors de l’exercice aérobie pratiqué comme activité principale. Ce qui aurait renforcé davantage la puissance de leurs membres inférieurs comme l’ont confirmé les travaux de Miyakate et al. [31] qui ont montré que les individus qui avaient l’habitude de s’exercer régulièrement avaient de meilleures performances de force de préhension et de puissance des membres inférieurs que ceux qui n’en avaient pas l’habitude.

Malgré un indice d’activité sportive significativement élevé en faveur des sujets encadrés, aucune différence significative n’a été enregistrée pour la pression artérielle diastolique (PAD) la pression artérielle systolique (PAS), le CT, les TG, le HDL, le LDL et la glycémie à jeun. Ces résultats sont consistants avec ceux des études antérieures. En effet, Dunn et al. [15] en comparant les effets induits par un programme lié au mode de vie actif à ceux d’un programme d’exercice traditionnel de 24 mois ont noté des améliorations comparables pour le ${\dot{\text{V}}\text{O}}_{\text{2}}\max$, le profil lipidique, la PAD, la PAS et le pourcentage de masse grasse sans modification de la masse corporelle chez des femmes et des hommes sédentaires. Subramanian et al. [16] n’ont pas également enregistré une différence significative pour les valeurs anthropométriques et la composition corporelle mais ont noté une différence significative (p < 0,05) pour le ${\dot{\text{V}}\text{O}}_{\text{2}}\max$, la pression artérielle en comparant les effets d’une activité physique régulière non structurée à ceux d’un entraînement sportif chez des adolescents. Beaucoup d’études ont indiqué que pour espérer des améliorations importantes des profils lipidique et anthropométrique, des changements de comportements alimentaires sont nécessaires aussi bien que l’accomplissement d’activités physiques intenses [32]. L’activité physique présentait une forte corrélation positive avec la force de préhension (r= 0,42, p < 0,001), la détente verticale (r= 0,42, p < 0,001) et l’endurance aérobie (${\dot{\text{V}}\text{O}}_{\text{2}}\max$) alors qu’elle était négativement corrélée avec la fréquence cardiaque de repos (r = -0,51, p < 0,01) dans le groupe encadré. Ces résultats vont dans le même sens que ceux d’Aadahl et al. [33] qui ont enregistré une forte corrélation positive entre la force de préhension, la puissance concentrique des membres inferieurs et l’activité physique de loisir. Huang et Malina [34] ont montré que l’activité physique était positivement corrélée à l’endurance aérobie chez les adolescents taïwanais. Cette relation inverse entre la fréquence cardiaque de repos et l’activité physique était attendue vu que la pratique régulière des activités physiques et sportives induit une libération accrue de l’acétylcholine, hormone impliquée dans l’abaissement de la fréquence cardiaque de repos. En effet, Jürgen [35] a constaté chez des sujets entraînés des taux d’acétylcholine (substance vagotonique ou parasympathique) nettement élevés et des taux de catécholamines (substance sympathique) réduits d’un tiers comparativement à ceux des sujets non entraînés. Fox et Mathews [36] ont aussi montré que l’inhibition du système sympathique entraîne une diminution de la fréquence cardiaque pouvant atteindre 30 battements par minute chez les sportifs confirmés. Or, l’abaissement de la fréquence cardiaque permet une réduction considérable du travail cardiaque conduisant à un risque réduit des maladies cardiovasculaires [37]. La corrélation enregistrée entre le niveau d’activité physique et les paramètres physiologiques d’une part et les paramètres métaboliques d’autre part était dans le sens attendu (excepté pour la glycémie à jeun) bien que non significative. Ces résultats sont en conformité avec ceux de Skoumas et al. [38] qui ont rapporté une association négative significative entre le niveau d’activité physique et les marqueurs de risque cardiovasculaire chez les femmes et non chez les hommes. Muhihi et al. [39] ont également rapporté une association négative non significative entre la dépense énergétique et les facteurs de risque de la maladie cardiovasculaire chez des jeunes hommes adultes. La raison principale suggérée par ces auteurs serait l’erreur qui peut être générée par l’utilisation d’un questionnaire, bien que valide, dans la mesure de ce comportement aussi complexe qu’est l’activité physique. La prévalence des facteurs de risque de la maladie cardiovasculaire et diabétique était moins élevée surtout chez les sujets encadrés exception faite pour le HDL bas. La recherche a montré que la sédentarité, une alimentation pauvre en graisse, certaines anomalies génétiques [40] ainsi qu’un manque de consommation d’alcool [41] peuvent être une source de basses concentrations de HDL. Cependant, les sujets de notre étude avaient un indice d’activité physique comparable à celui d’autres populations physiquement actives [42] mais plus de 60% d’entre eux avaient déclaré s’abstenir de la consommation d’alcool.

Il a été démontré qu’un état de bonne condition physique et/ou un mode de vie actif sont associés à une réduction considérable du risque de maladies cardiovasculaires [43]. Plusieurs mécanismes biologiques à travers lesquels l’activité physique diminue le risque des maladies cardiovasculaires ont été postulés notamment l’abaissement des niveaux de la pression artérielle [44, 45], les marqueurs de l’inflammation et de la coagulation [46, 47]. Cette étude avait des limites. Les sujets qui y ont participé étaient moyennement âgés et n’avaient probablement pas encore développé beaucoup de facteurs de risque des maladies chroniques non transmissibles. En outre, c’était une étude transversale qui ne peut pas bien établir des liens de causalité entre l’activité physique et les paramètres de la condition physique liée à la santé.

## Conclusion

Le niveau d’activité physique des burundais qui bénéficient d’un encadrement professionnel est plus élevé que celui des adultes qui n’en bénéficient pas. Comparés aux adultes encadrés, les sujets non encadrés ont des lacunes aux niveaux musculaire et physiologique. Néanmoins, bien que ce ne soit pas à un niveau comparable, les deux groupes présentent un bon profil des facteurs de risque de maladies cardiovasculaires. La pratique d’activités physiques et même la pratique populaire spontanée, induisent donc des adaptations de l´organisme favorables à la santé de la population urbaine. Cependant, en vue de maintenir ou d’améliorer sa santé, cette population devrait élever son niveau d’activité physique compte tenue de la transition nutritionnelle en cours et de l’urbanisation rapide. En outre, elle devrait être encouragée à associer le renforcement musculaire à l’exercice aérobie étant donné que beaucoup d’activités de la vie quotidienne dépendent d’une ou plusieurs des composantes de la condition musculo-squelettique surtout chez les personnes vieillissantes. Enfin, une étude longitudinale et expérimentale sur des adultes plus âgés ou en surpoids est nécessaire pour bien appréhender le rôle de l’activité physique d’entretien dans l’amélioration de la condition physique liée à la santé dans notre environnement.

### Etat des connaissances actuelles sur le sujet

- L'activité physique régulière influence positivement la condition physique liée à la santé dont plusieurs composantes sont considérées comme des précurseurs de la maladie et identifiées comme des facteurs de risque en recherche clinique.

### Contribution de notre étude à la connaissance

- L’activité physique libre est aussi efficace que l’activité physique encadrée dans le maintien d’une bonne condition physique et d’un bon profil cardiovasculaire bien que l’activité physique encadrée apporte des bénéfices supplémentaires;
- Dans nos pays en développement où le manque de moyens financiers limite les inscriptions dans les salles de sports privées, l’activité physique libre devrait être encouragée dans le cadre de la lutte contre les maladies liées au mode de vie sédentaire;
- Cependant, elle doit intégrer le renforcement musculaire qui est souvent reléguée à l’arrière-plan.
